# Surgery for diverticular peritonitis

**DOI:** 10.3389/fmed.2025.1501734

**Published:** 2025-02-11

**Authors:** Savvas Papagrigoriadis, Anestis Charalampopoulos

**Affiliations:** ^1^IASO General Hospital, Athens, Greece; ^2^OneWelbeck Digestive Health Centre, London, United Kingdom; ^3^Medical School, 3rd Surgery Unit, Attikon University Hospital, National and Kapodistrian University of Athens, Athens, Greece

**Keywords:** diverticulitis, peritonitis, Laparoscopic Peritoneal Lavage, diverticular disease, colostomy, Hinchey, diverticular abscess, emergency laparoscopy

## Abstract

Some patients with acute diverticulitis will present with colonic perforation and peritonitis. This paper is a review of the surgical management of diverticular peritonitis Hinchey III and Hinchey IV. The significance of prompt management of sepsis is discussed. The surgical options for Hinchey III and Hinchey IV peritonitis are discussed with presentation of the supporting literature. In Hinchey III peritonitis Laparoscopic Peritoneal Lavage has emerged as an alternative to laparotomy—colectomy. The classic Hartmann’s operation has no advantage of survival and results frequently in permanent stoma. Recent published evidence supports on table colonic lavage and the performance of primary anastomosis unless the patient is critically ill.

## Introduction

A significant percentage of patients with diverticulitis present with clinical peritonitis. This is the group of patients at highest risk of mortality, Intensive Care hospitalisation and formation of permanent stoma. The treatment of those patients is challenging and guidelines are required the most. According to traditional teaching peritonitis is inflammation of the peritoneum (1). Peritonitis can be either localised or generalised, involving the entire abdomen.

### General management of peritonitis

The clinical diagnosis of peritonitis relies on the classical symptoms of

- Pain, this is sharp and constant. Pain might be localised initially and become generalised when the peritonitis spreads in the entire abdomen.
- Nausea, vomiting, fever may be present
- On abdominal examination there is abdominal tenderness, abdominal wall guarding and rigidity, rebound tenderness of the peritoneum.
- The features of *sepsis* may be present in clinical observations and laboratory values (2):
  The *Systematic Inflammatory Response (SIRS) Criteria*: Temperature > 38, Heart rate > 90, Respiratory rate > 20, White Blood Cells >12,000 or < 4,000.
  If a suspected source of infection (in this case diverticulitis) is also present then the patient is *suffering from sepsis.*
- There should be immediate investigation as to whether there are any *severe sepsis criteria* > Lactic acidosis, Systolic Blood Pressure < 90 mm Hg or drop >40 mm Hg.
- If there is also hypotension then the patient is in *septic shock*.
- If there is evidence of 2 or more organs failing, then the patient is in *Multiple Organ Dysfunction Syndrome* Criteria.

When a patient with diverticular peritonitis is assessed then the above diagnostic process and classification is essential prior to anything else. The most important urgent task is the determination of how septic, effectively how ill, the patient is. If the patient is found to be in sepsis, then immediate steps have to be taken to face sepsis, the management of which is time-sensitive. Those urgent measures are memorised in the table of “*The Sepsis Six*” (3):

- Give O2 to keep SATS above 94%
- Take blood cultures
- Give IV antibiotics
- Give a fluid challenge
- Measure Lactate
- Measure Urine Output

The above diagnostic and therapeutic actions should be performed without delay on all patients with suspected peritonitis form diverticulitis. The diagnosis of peritonitis is primarily a clinical diagnosis. In recent years a clinical diagnosis of peritonitis beyond doubt signified the immediate surgical procedure, *exploratory laparotomy* in order to both diagnose the cause and provide surgical treatment.

If a patient is anuric and hypotensive then an aggressive attempt should be made to resuscitate the blood pressure, circulation and renal function before the laparotomy. Crystalloids and electrolytes should be administered as per requirement. Antibiotics have to be started immediately. It has been demonstrated in studies that delay in administration of antibiotics increases mortality (4).

### Laparoscopy

The presence of pneumoperitoneum on the plain abdominal x ray is evidence of viscus perforation. In the presence of a clinical picture of peritonitis many surgeons would take the patient to the operating theatre without a CT scan. If the patient is stable there is never such a thing as “too much information” prior to exploratory laparotomy. The amount and location of the free air can give a clue as to the source. For example, a thin slice of air under the diaphragm is more common with gastroduodenal perforations while a large amount of air, and especially if it is under both diaphragms is a common finding of colonic perforation.

A diagnostic laparoscopy can always be performed at no time expense in all emergency operating theatres nowadays (5). As there is commonly abdominal distension the preferred method of the pneumoperitoneum is the “open Hasson technique” and not the Veress needle. If a midline prior incision exists, then introduction of the Hasson’s port in the left upper quadrant is the preferred option by many.

Once the camera and one more 5 mm port is in usually the diagnosis of peritonitis is immediately obvious. If there is only pus then this is Hinchey III peritonitis. If there is faecal matter then it is Hinchey IV.

The practical difference between the two is:

If it is Hinchey III then a thorough look for the perforation has to be made. If a hole is identified then conversion to sigmoid colectomy (including the hole) has to take place.

If a perforation is not identified then the planning of the margins of the colonic resection becomes somewhat difficult and it will probably end up in an extensive left colectomy and not just a sigmoid colectomy (as most surgeons will prefer to be) “on the safe side.”

This kind of circumstance generated the idea and the concept of “Laparoscopic Peritoneal Lavage.” We will explore that before we discuss faecal peritonitis.

### Laparoscopic peritoneal lavage

This is literally a washout of the peritoneal cavity via a laparoscopy. Why do it? Because in the absence of an identifiable perforation a colectomy (a) may be difficult to plan (b) may not be immediately necessary.

By “not immediately necessary” we imply that the colectomy may be required at a later stage. Why think that? Why not just get on with it, do the colectomy and give a definitive solution? The answer lies in the extent of systemic illness of the patient at that particular moment. Many patients are too ill for a major operation because of systemic sepsis at the time. Even though some inexperienced surgeons believe they can do relatively harmless “quick Hartmann’s” the literature actually shows that Hartmann’s in the septic patient has high morbidity and mortality, surprisingly even higher than a colectomy with an anastomosis (6). The other issue is the need for subsequent surgery for stoma reversal. Experience shows that, for a variety of reasons, a considerable number of patients never have their end colostomy reversed (7).

The performance of LPL offers an interim solution, a “bridge strategy” until there is a decision for definitive colectomy. However, even since the very first reports it was highlighted that many patients may never require a colectomy after the LPL (8, 9). Myers et al. (10) reported in 2008 a series of 92 patients managed by LPL. Two of the patients required postoperative drainage of abscess and there was 3% mortality. Only 2 patients represented with diverticulitis at a median follow up of 36 months.

Liang et al. compared LPL versus laparoscopic Hartmann’s (LHP) in generalised peritonitis (11). They found that only 44% of those who had LPL proceeded to sigmoidectomy later. LPL had better short and long term outcomes than LHP.

The DILALA trial (12) compared laparoscopic lavage with resection as treatment for perforated diverticulitis. Patients with laparoscopic lavage had a 45% reduction of risk to undergo colectomy and colostomy at 2 years. There was no other difference.

A systematic review on LPL for Hinchey III peritonitis by Scarpinata and Papagrigoriadis (13) on 230 patients by 10 studies found that there was only 1.3% failure rate of LPL, morbidity 12.7% and no mortality. Only 2.6% of patients got readmitted after LPL and only 0.8% required surgery.

A study on long-term outcomes of Laparoscopic Peritoneal Lavage by Campana et al. on 69 patients who had LPL as far back as 15 years, found a long-term of recurrence of 42%. Smoking and episodes of diverticulitis prior to LPL were risk factors for recurrence (14).

Not all authors are in support of LPL. Penna et al. consucted a systematic review of LPL versus colonic resection in 589 patients with Hinchey III from 7 studies (3 randomised) (15). No significant differences were noted for mortality, 30-day reoperations and unplanned readmissions. LL had higher rates of intraabdominal abscesses (POR¼ 2.85; 95% confidence interval, CI, 1.52–5.34; P¼ 0.001), peritonitis (POR¼ 7.80; 95% CI 2.12–28.69; P¼ 0.002), and increased long-term emergency reoperations (POR¼ 3.32; 95% CI 1.73–6.38; *p* < 0.001). Benefits of LL included shorter operative time, fewer cardiac complications, fewer wound infections, and shorter hospital stay. LPL had shorter operative time, fewer cardiac complications, fewer wound infections, and shorter hospital stay. On the issue of stoma, 90% of the colonic resection patients had a stome (temporary or permanent) versus only 14% in LPL. Of the LPL patients 36% had an elective sigmoidectomy at a second stage. The authors concluded that LPL was associated with a higher risk of persistent peritonitis.

The present authors of this review believe that the above results are consistent with the design of the LPL strategy which is primarily as a “bridge procedure” with a view to minimise the risks of colonic resection during peritonitis and avoid the high stoma formation rates. It is expected that many LPL patients will require an elective second stage procedure because of persistent inflammation. The results of the Penna wet al review are not dictating a strategy against LPL but rather an individualised approach per each patient.

The Penna systematic review includes a majority of non-randomised trials which naturally includes patients with great heterogeneity of clinical circumstances. Another systematic review by Marshall et al. (16) was confined in only 3 randomised trials of LPL versus colonic resection in Hinchey III peritonitis and found a higher re-intervention rate in LPL (28.3% v. 8.8%) but no difference in ICU admission, mortality or 12—month stoma rate.

It therefore seems that LPL can be an efficient definitive treatment for diverticular peritonitis Hinchey III in some cases but there is also a significant group, probably one-third of patients, where a subsequent elective operation may be required.

The difference in several studies on that point is likely to be due to application of different selection criteria for LPL.

However, the systematic reviews that have highlighted the high rate of reinterventions in LPL have also found that in the majority of patients a reintervention has not been required. Therefore even if it is not a universal change of strategy, LPL remains a valid alternative option to be discussed with the patient. This is consistent with the epidemiological observation that it is generally the first attack of diverticulitis which is the most life—threatening.

From the technical point of view LPL should not be underestimated as an “easy” procedure which can be undertaken by an inexperienced on-call junior surgeon. Very often the small bowel loops form a mass surrounding and adherent on the sigmoid colon site of perforation. This mass needs to be carefully and gently dissected with the utmost attention to avoid tears of the serosa which can create holes. In addition, the small bowel loops in the rest of the abdomen are often adherent amongst themselves with pseudo-membranes and contain small abscesses (interloop abscesses). Interloop abscesses can be missed on CT scan and need to be identified and drained for resolution of the sepsis. Following drainage of all visible pus there is thorough washout of all quadrants with copious amounts of warm saline, at least four litres are required. It is important to wash above the liver and the spleen to avoid subsequent subphrenic collections.

The laparoscopy may take a considerable length of time, one hour is not unusual, to ensure complete drainage and adequate irrigation. Two abdominal drains are usually placed although there is no evidence that they prevent recurrence of collections and they are probably unnecessary if the drainage and irrigation have been thorough. The placement of drains should not be used as compensation for lack of meticulous dissection-drainage-irrigation. It is obvious that the procedure needs to be performed by a surgeon who is experienced in laparoscopy.

LPL is not a day case laparoscopy. It requires hospitalisation until firstly the abdominal sepsis and then the complicated diverticulitis that has caused it, have both subsided. The systematic review by Scarpinata and Papagrigoriadis have found a hospital stay ranging from 7 to 14 days with a mean of 6.6 days. That was significantly shorter than Hartmann’s which had a mean of 16 days.

More encouraging was the low readmission rate (only 2.6%) which persisted through long follow up between 6 and 96 months.

Even though there are no randomised trials on this point, there are reports of complementary percutaneous drainage under CT scan guidance of recurrent small collections with good results. A prerequisite is that the patient is in good clinical condition.

It therefore seems that the majority of patients with Hinchey III who have LPL avoid the sigmoid colectomy and also a stoma, either ileostomy or colostomy.

However, it should be noted that LPL is not a treatment which is applied to all patients, there is a need for discretion, clinical judgement and constant monitoring of the patient’s condition.

The ideal patient for LPL would be someone without severe comorbidities so that they are not frail and without organs and systems especially vulnerable to sepsis. That candidate patient should be someone without prior complex surgery of the lower abdomen that may have adhesions that would make the laparoscopy challenging.

If the patient fails to improve following LPL a sigmoid colectomy can be performed in the same hospitalisation. If there is early recurrence of the diverticulitis and readmission then the sigmoid colectomy can be performed at a second stage. If it is applied under those conditions then LPL is safe and very useful in improving morbidity, mortality, and stoma rates in Hinchey III diverticular peritonitis.

## Surgery for faecal peritonitis—Hinchey IV

Faecal peritonitis is one of the most dire abdominal emergencies with a very high mortality, especially if it is not timely treated.

Emergency admission for acute diverticulitis carries a significant risk of death, and practically all of those deaths concern patients with diffuse peritonitis which results in generalised sepsis. A USA study found that the overall mortality of emergency admissions for acute diverticulitis was 5.1% but that figure depends a lot on the risk factors of the patient. The factors associated with death were age, functional class, dyspnoea, ascites, ASA class, prior radiotherapy, corticosteroid use, creatinine levels, albumin levels. Patient risk increased with the number of factors per case, if they had 4 factors the risk of death was 53%. Therefore the clinical assessment of the patient on admission is of vital importance because the level of risk will dictate the urgency of all the therapeutic actions.

There have been concerns that the Hartmann’s procedure does not actually protect from mortality. A systematic review by Constantinides et al. on 963 patients did not reveal any difference in mortality in peritonitis Hinchey III and IV between primary anastomosis and Hartmann’s.

A randomised study by Binda et al. found no difference in mortality between primary anastomosis and Hartmann (17). A study from Switzerland found lower mortality with primary anastomosis as well (18), so did a study from France (19). Both studies found that when a temporary ileostomy was formed the rate of permanent stoma was much lower than with Hartmann’s.

The DIVA arm of the LADIES trial found no difference in morbidity or mortality between primary anastomosis and Hartmann’s (20). The decision for temporary ileostomy was discretional to the surgeon and had no impact either. The stoma closure rate after ileostomy was better than that of end colostomy. They concluded that in haemodynamically stable, immunocompetent patients younger than 85 years, primary anastomosis is preferable to Hartmann’s procedure as a treatment for perforated diverticulitis (Hinchey III or Hinchey IV disease). A meta-analysis in 2018 demonstrated a lower rate of surgical infections for primary anastomosis without increased morbidity or mortality (21). A number of meta-analyses and randomised trials have supported the above data with overwhelming repetition of the findings (22, 23).

The evidence in favour of the primary anastomosis is such with this abundance of randomised studies and meta-analysis, that there has been a declaration of the “The end of the Hartmann’s era for perforated diverticulitis” (24).

From the operating and technical point of view there are factors to consider when the time for implementation of those findings comes. The experience of the operating surgeon should be adequate to exercise judgement on the clinical decisions in theatre and to be able to perform the primary anastomosis technically. The experience of the anaesthetist should also be of the same level. It should be emphasized that a patient with faecal peritonitis is one of the most at risk patients a general surgical department can deal with, and the highest level of skills and experience have to be recruited. The old-fashioned practice to hand over the case to junior on-call surgeons for a “quick Hartmann’s” in the middle of the night is not acceptable today.

With regards to the choice between laparoscopy and laparotomy this is a decision of the surgeon. While laparoscopy has become the current gold standard for Hinchey I, II, III elective or urgent diverticulitis surgery, Hinchey IV faecal peritonitis requires that technique retreats into second priority and the first priority is the achievement of the haemodynamic stability and the reversal of sepsis. Hence the responsibility as to the technique lies with the surgeon and their insight of their own skills and capabilities. This author would feel comfortable doing a laparoscopic sigmoid colectomy on a stable patient with Hinchey III purulent peritonitis but would be sceptical to apply the same on a hypotensive, tachycardic, septic patient with faecal peritonitis. The very formation of pneumoperitoneum and the required Trendelenburg tilt of the operating table might risk excessive strain on the heart and the lungs under those circumstances.

Thorough washout with warm saline of all faecal material is immediately performed on entering the abdomen. Two powerful large bore suctions are used with concurrent provision by the scrub nurse of multiple “buckets” of warm saline. Diluted betadine solution is used by some but there is no evidence that it is superior to saline. At the same time the perforation of the colon is identified and two soft bowel clamps can be placed to stop leakage of faecal matter. Alternatively, an overrunning silk or Vicryl suture can be used to close the hole temporarily, but not much time should be spent on this as the priority is the irrigation. Once all quadrants of the abdomen, the pelvis and the diaphragmatic spaces have been irrigated then the planning of the operation takes place. Full mobilisation and washout of all the small bowel loops is also performed.

The first step is dissection of any small bowel loops which may form a mass over the perforation.

In the event of non-identification of the perforation, which can happen if it is 2 or more days old, the colectomy is planned on the grounds of the inflamed part of the colon. It usually is easily identifiable as a thickened mass which is surrounded by thickened colon distally and centrally. The mesocolon is also thickened and bleeds easily on dissection. The resection has to be made on healthy margins on either side. Healthy means the lack of inflammation, but it is often the case that diverticula may extend into the descending colon or even in the transverse and right colon. There should be no resection of the bowel and anastomosis through diverticula orifices (for the risk of leak) but the extension of the resection to include all visible diverticula is not advisable. It is still a mystery with many theories why most of the diverticulitis happens in the sigmoid colon, even though there may be diverticula on the entire colon.

The resection is commonly on the junction between the sigmoid and descending colon and distally just above the peritoneal reflection. Leaving a distal part of the sigmoid is considered to increase the risk of recurrent diverticulitis.

There has been debate about the necessity of the ligation of the inferior mesenteric artery the argument being that sparing it may reduce the risk of anastomotic leak. However this has not been confirmed in studies and therefore it is left to the judgement of the surgeon (25). Many surgeons feel that ligation of the inferior mesenteric artery allows for a better blood-free surgical dissection plane and they prefer it.

In the event of performing a left hemicolectomy in order to include an inflamed part of the descending colon additional care should be taken to check the patency of the marginal artery and the meandering artery which ensure the vascular supply of the splenic flexure. This will decrease the risk of anastomotic leak.

The mobilisation of the splenic flexure is thought to increase the ease of approximating the ends for anastomosis without tension and thus decreasing the risk of anastomotic leak (26). Spending an additional 15 min for splenic flexure mobilisation is worth it for that reason.

At the time of the anastomosis the condition of the colon is considered. It is crucial that both ends are viable, with good blood supply as deducted by the bleeding from the divided edges.

The anastomosis can be performed either hand sewn or stapled -there is tons of literature on this issue and all studies conclude that there is practically no difference in the outcomes attributable to the technique. The author finds the introduction of the circular staple from below in the distal sigmoid slightly risky for tear of the rectum. If there is a long rectal stump, then it may be more convenient to perform a hand sewn descending-rectal anastomosis. The anastomosis can be performed in one or two layers and there is a variety of suture choices, the author uses PDS 2/0 or 3/0.

The decision for a temporary ileostomy is left to the surgeon. In contrast to the elective resection for Hinchey I and II (where the guidance is not to do an ileostomy), if there is heavy inflammation of the colon then an ileostomy will be chosen by many surgeons. An obvious problem however is that in unprepared bowel (as is the case in faecal peritonitis) the ileostomy cannot do anything about the faecal load of the colon, hence its preventive powers are very limited. The proposed solution is the *“on table lavage”* i.e., a washout of the colon. This is performed via either an enterotomy on the caecum or, preferably via an appendicectomy and introduction of a Foley catheter through the opening. Antegrade irrigation of the colon with copious saline is performed (27). The procedure is done before the anastomosis and the proximal colon is placed in dishes for emptying of the faecal matter. The process is messy and results in inevitable contamination of the abdomen and the wound, and for that reason some surgeons are against it. There is concern that the practice may be increasing the risk of surgical wound infections. The argument is that lack of bowel preparation and omission of lavage does not actually increase anastomotic leak rates in any of the studies that looked into this question. On table lavage takes time, probably between 15 and 30 min, and therefore the prerequisite is that the patient is stable and able to tolerate the extra general anaesthetic time. This issue is not likely to be resolved in randomised trials and either of the two practices is acceptable.

Once the anastomosis is performed it is checked via the “classic” tire test. That consists of filling the abdominal cavity with saline and inflating the rectum with air via a foley catheter. The presence of bubbles means the anastomosis is not air-tight, the site of leak is identified and over sutured with PDS or Vicryl, and a new tire test is performed.

At the end of the operation if the patient is well enough to be extubated and not in need of inotropes, then they can be transferred to the regular word for standard monitoring. If there are any concerns about ventilation or circulation support, then the patient can be transferred to the Intensive Care Unit in state of ventilation for intense monitoring and the extubation can be postponed for the next few hours or the next day.

A nasogastric tube is not kept in after extubation, according to enhanced recovery protocols. Antibiotics are given intravenously for 7 days. The most commonly used are synthetic penicillins such as tazobactam, second or third generation cephalosporins, or quinolones in order to cover gram negative and gram-positive bacteria. As per all colonic surgery cover for anaerobic organisms is required and metronidazole is additionally provided.

### Outcomes of laparoscopic surgery for Hinchey III & IV peritonitis

Lee et al. (28) published a study on the database of the American college of Surgeons in which 3,299 open operations of perforated diverticular peritonitis were compared with 282 laparoscopic- completed cases. There were also 175 cases of conversion to open. The laparoscopic - completed approach had better outcomes than the open surgery, i.e., fewer complications, less unplanned intubation and acute renal failure. The advantages of laparoscopy remained even if the case had to be converted to open.

Cassini et al. (29) examined 60 patients with diffuse diverticular peritonitis Hinchey III and IV who had either Laparoscopic (36) or open (24) surgery. Laparoscopic patients had around half the incidence of complications of the open one while the mortality was high in both groups, around 16%. As this was not a randomised trial it cannot serve as guidance, it is likely that the sicker patients may have gotten open surgery.

Mathematical model analysis for the optimal operative strategy for Hinchey III sigmoid diverticulitis by Dossa et al. (30) concluded that primary resection and anastomosis is the optimal approach yielding higher quality-adjusted life years.

A particular case of complicated diverticulitis is when it involves colovesical fistulas, which are a technically challenging variance. A systematic review by Cirocchi et al. (31) examined 25 studies and 202 patients with laparoscopic resection and primary anastomosis of colovesical fistulas. There was zero mortality and only one anastomotic leak. The authors concluded that laparoscopic surgery was feasible even for those complex cases.

A meta-analysis by Horesh et al. examined the long-term outcomes of surgical treatment of perforated diverticulitis (32). Five randomised trials with 499 patients were analysed. In three of the studies Laparoscopic Lavage was found to be better in terms of long-term stoma and reoperation compared to colonic resection. However LPL had higher odds (OR-5.8) of disease recurrence. Another two randomised studies examined the performance of colectomy with primary anastomosis compared to Hartmann’s. The primary anastomosis offered lower risk of long-term stoma, fewer long-term complications. Primary anastomosis carried also a lower incidence of incisional hernia and reoperation.

This meta-analysis indicates that LPL should always be considered taking into account the higher risk of recurrence.

## Conclusion

Diverticular peritonitis is a life-threatening condition which requires management according to the general guidelines of abdominal sepsis. In the past the standard approach was laparotomy with Hartmann’s sigmoid colectomy and end-colostomy. Recent evidence suggests that there is value in using laparoscopic techniques to distinguish between Hinchey III peritonitis with pus contents and Hinchey IV faecal peritonitis. In selected patients Hinchey III peritonitis can be treated with LPL either as a bridge procedure or a sole treatment but it requires experienced and skilled surgeons. The choice of surgery for faecal peritonitis Hinchey IV depends primarily on how critical the condition of the patient is. There is the choice of either a Hartmann’s procedure or of a primary anastomosis with prophylactic ileostomy provided that the patient is stable and the surgeon is a specialist in colorectal surgery.
